# Late-Onset Tay-Sachs Disease With a Predominantly Neuropsychiatric Presentation: Case Report and Literature Review

**DOI:** 10.1192/bjo.2024.696

**Published:** 2024-08-01

**Authors:** Graeme Yortston, Noor Ul Ain Awan, Mahmoud Aref, Srinivasa Thirumalai

**Affiliations:** St Matthew's Healthcare, Northampton, United Kingdom

## Abstract

**Aims:**

Background: Late-onset Tay-Sachs disease (LOTS) is an autosomal recessive lysosomal storage disease due to a variety of mutations in the hexosaminidase-A gene which leads to accumulation of GM2 ganglioside in the brain. It typically presents in late adolescence with a slowly progressive spectrum of neurologic symptoms including lower-extremity weakness with muscle atrophy, dysarthria, incoordination, tremor and mild spasticity and/or dystonia. Psychiatric symptoms including mood disorder, psychosis and neurocognitive symptoms occur in around 50% of cases but are rarely the presenting feature.

**Methods:**

Case Report: Patient X is a 35 year old man of Irish descent currently detained in an independent hospital locked rehabilitation unit following the breakdown of a care home placement. He first presented to mental health services at the age of 17 with psychomotor agitation, rapidly changeable moods, manic-like symptoms and sexual disinhibition. He was diagnosed with schizoaffective disorder, attention deficit hyperactivity disorder and Asperger's syndrome and he had several compulsory hospital admissions over the next five years before a prolonged period of rehabilitation and discharge to a residential home for people with autistic spectrum disorders. However, he continued to exhibit disruptive behaviour, often triggered by periods of insomnia and had further hospital admissions. When he was 31 his brother was diagnosed with LOTS and this led to him being tested and found to have the same mutation.

**Results:**

Discussion: There had been no suspicion of a neuropsychiatric disorder prior to the diagnosis of the patient's brother with LOTS and he was treated with conventional psychotropic medication with limited success. However, when the case records were obtained from his first hospital admission there was evidence of dysarthria although the significance of this was not appreciated. With hindsight many of his other symptoms can be seen as indicative of a neuropsychiatric disorder.

**Conclusion:**

It is important to take a family history and consider a neuropsychiatric condition in families with multiple affected individuals. There are as yet no specific treatments for LOTS, and management is aimed at symptom reduction and enhancing quality of life, but a number of disease modifying strategies are being investigated including enzyme replacement therapy, pharmaceutical chaperone therapy, substrate reduction therapy, gene therapy, and hematopoietic stem cell replacement therapy, making it even more important the condition is recognized early.

